# Trial Forge Guidance 3: randomised trials and how to recruit and retain individuals from ethnic minority groups—practical guidance to support better practice

**DOI:** 10.1186/s13063-022-06553-w

**Published:** 2022-08-17

**Authors:** Shoba Dawson, Katie Banister, Katie Biggs, Seonaidh Cotton, Declan Devane, Heidi Gardner, Katie Gillies, Gosala Gopalakrishnan, Talia Isaacs, Kamlesh Khunti, Alistair Nichol, Adwoa Parker, Amy M. Russell, Victoria Shepherd, Frances Shiely, Gillian Shorter, Bella Starling, Hywel Williams, Andrew Willis, Miles D. Witham, Shaun Treweek

**Affiliations:** 1 Centre for Academic Primary Care, Bristol Medical School, University of Bristol, Bristol, BS8 2PS UK; 2 Health Services Research Unit, University of Aberdeen, Aberdeen, AB25 2ZD UK; 3 School of Health and Related Research, University of Sheffield, Sheffield, S1 4DA UK; 4 Health Research Board-Trials Methodology Research Network (HRB-TMRN), School of Nursing and Midwifery, National University of Ireland Galway, University Road, Galway, Ireland; 5 Department of Surgery and Cancer, Imperial College London, London, W12 0NN UK; 6 UCL Centre for Applied Linguistics, IOE, UCL’s Faculty of Education and Society, University College London, London, WC1H 0AL UK; 7 Diabetes Research Centre, College of Life Sciences, University of Leicester, Leicester General Hospital, Leicester, LE5 4PW UK; 8 National Institute for Health Research (NIHR), Applied Research Collaboration (ARC) East Midlands, University of Leicester, Leicester, UK; 9 School of Medicine, University College Dublin, Dublin, Ireland; 10 York Clinical Trials Unit, University of York, York, UK; 11 WHO Disability Team, Geneva/ Leeds Institute of Health Sciences, University of Leeds, Leeds, UK; 12 Centre for Trials Research, Cardiff University, Neuadd Meirionnydd, Heath Park, Cardiff, CF14 4YS UK; 13 Health Research Board Clinical Research Facility and School of Public Health, University College Cork, Cork, Ireland; 14 Drug and Alcohol Research Network, Queen’s University Belfast, Belfast, UK; 15 Centre for Improving Health Related Quality of Life, School of Psychology, Queen’s University Belfast, Belfast, UK; 16 Public Programmes Team (now Vocal), Manchester University NHS Foundation Trust, Research & Innovation Division, The Nowgen Centre, 29 Grafton Street, Manchester, M13 9WU UK; 17 NIHR Manchester Biomedical Research Centre, NIHR Manchester Clinical Research Facility, Manchester, UK; 18 Centre of Evidence-Based Dermatology, Queen’s Medical Centre, Nottingham University Hospitals NHS Trust, Nottingham, NG7 2UH UK; 19 NIHR ARC East Midlands, University of Leicester, Leicester, UK; 20 NIHR Newcastle Biomedical Research Centre, Campus for Ageing and Vitality, Newcastle University and Newcastle upon Tyne NHS Trust, Newcastle, NE4 5PL UK

**Keywords:** Randomised trials, Ethnic minority, Recruitment, Retention, Inclusion, Trial methodology

## Abstract

Randomised trials, especially those intended to directly inform clinical practice and policy, should be designed to reflect all those who could benefit from the intervention under test should it prove effective. This does not always happen. The UK National Institute for Health and Care Research (NIHR) INCLUDE project identified many groups in the UK that are under-served by trials, including ethnic minorities.

This guidance document presents four key recommendations for designing and running trials that include the ethnic groups needed by the trial. These are (1) ensure eligibility criteria and recruitment pathway do not limit participation in ways you do not intend, (2) ensure your trial materials are developed with inclusion in mind, (3) ensure staff are culturally competent and (4) build trusting partnerships with community organisations that work with ethnic minority groups. Each recommendation comes with best practice advice, public contributor testimonials, examples of the inclusion problem tackled by the recommendation, or strategies to mitigate the problem, as well as a collection of resources to support implementation of the recommendations.

We encourage trial teams to follow the recommendations and, where possible, evaluate the strategies they use to implement them. Finally, while our primary audience is those designing, running and reporting trials, we hope funders, grant reviewers and approvals agencies may also find our guidance useful.

**Supplementary Information:**

The online version contains supplementary material available at 10.1186/s13063-022-06553-w.

## Background

Randomised trials, especially those intended to directly inform clinical practice and policy, should be designed to reflect all those who could benefit from the intervention under test should it prove effective. This does not always happen. The UK National Institute for Health and Care Research (NIHR) ‘Innovations in Clinical Trial Design and Delivery for underserved groups’ (INCLUDE) project, initiated in 2017, identified many groups in the UK that are under-served by trials and by the healthcare services that follow from them [1]. INCLUDE is clear that trials need to widen inclusion and improve participation amongst these under-served groups.

One of these under-served groups is ethnic minorities. There is plenty of evidence that ethnic minority groups are under-represented in trials [2–5]. INCLUDE, Trial Forge (https://www.trialforge.org) and others have recently developed the INCLUDE Ethnicity Framework (https://www.trialforge.org/trial-forge-centre/include/), which helps trial teams to think about how disease, intervention and design influence the ability of people from different ethnic backgrounds to take part in a trial [6]. The aim is to make it more likely that trial results are widely applicable and acceptable to all those who could benefit. Early evaluation of the INCLUDE Ethnicity Framework is ongoing, but initial signs are that while the framework is not perfect and is likely to need some modifications, it is a useful tool to highlight trial inclusion issues linked to ethnicity.

This still leaves trial teams with the conundrum of what to do about the issues raised by the Ethnicity Framework. At present, there is little robust evidence to help trial teams to effectively recruit [7] and then retain [8] ethnic minority participants. In the absence of a robust evidence base, this Trial Forge Guidance aims to give recommendations, advice, testimonials, examples and resources to help trial teams improve the inclusion and retention of individuals from ethnic minority backgrounds in trials. Our primary audience is researchers, i.e. those designing, running and reporting trials. Others, including funders, grant reviewers, those disseminating trial results, members of ethics committees and organisations focusing on minority ethnic groups may also find the guidance useful. We have a UK focus, but we think the guidance is likely to have relevance beyond the UK.

We recognise that this guidance will need to be updated as more evidence becomes available and we commit to keeping the guidance up-to-date for at least the next 5 years. Ongoing work within Trial Forge, INCLUDE and the Medical Research Council-NIHR Trial Methodology Research Partnership (https://www.methodologyhubs.mrc.ac.uk/about/tmrp/) means that we envisage an update will be required within two years from publication.

## Improving recruitment and retention of ethnic minority participants

Trials rarely have explicit eligibility criteria that exclude ethnic minority groups but a combination of other factors often makes it much less likely that ethnic minority individuals will be able and willing to participate in a trial. We considered the existing literature on this topic area, combined with our experience of recruiting and retaining people from ethnic minority groups and trials, to identify and develop recommendations to improve the recruitment and retention of ethnic minority trial participants. Additionally, we presented these recommendations to public contributors from diverse ethnic backgrounds and sought their opinions.

We have made four recommendations because we think that they target issues that are without doubt important if trial teams are to successfully include ethnic minority individuals in their trials. There are other things that will affect recruitment and retention of ethnic minority individuals (e.g. health literacy and the perceived value of health research) but these affect other groups too. Other intersecting factors influence equity-relevant trials; some of these are highlighted in the PROGRESS PLUS framework (place of residence, race/ethnicity/culture/language, occupation, gender, religion, education, socio-economic status, social capital and ‘Plus’, which includes other context specific factors [9]. These factors have been considered as confounders or modifiers in health research but have been used much less to explore inequities in intervention studies [9].

For each of these recommendations, we also provide testimonials from public contributors (see Acknowledgements), advice for good practice and resources to help implementation. Other authors have made recommendations in this area (e.g. Bodicoat and colleagues [10]), and we do not disagree with their suggestions. However, here, we propose a short-list of four recommendations in the hope that progress might be faster if trial teams put substantial effort into following a small number of unequivocally important recommendations rather than trying to spread their limited resources across a long list of challenges.

At the outset of trial planning, a trial team needs to understand, in detail, the ethnicity of those affected by the disease being targeted by the trial because these are the people whose perspectives need to be reflected in trial design and conduct. Trials research does not always benefit ethnic minority individuals as trial teams do not always include ethnic minority groups in the design of the trial (as public contributors) or as participants [1]. Inclusive patient and public involvement is a prerequisite when designing and conducting trials to reflect the needs of everyone that the trial could potentially benefit. This is true for all trials but is absolutely essential for trials that aim to be of immediate clinical and policy relevance. Trial teams need to set targets for recruitment and retention of different ethnic groups and monitor progress against those targets, which means trial teams need to collect data on participants’ ethnicity. At present, most trials do not collect and report ethnicity data [11]. Even for COVID-19 where the importance of ethnicity is not in doubt, a recent review found only 34 of 209 preprints reported ethnicity data [12]. While ethnicity is a complex construct and can be challenging to measure, we strongly recommend trial teams collect, monitor and report ethnicity data for their trial population.

**Fig. 1:**
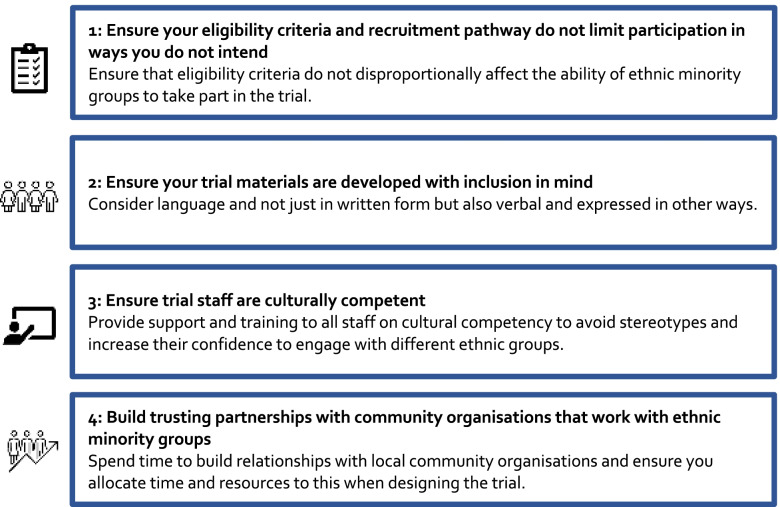
Four recommendations for designing and running trials that include the ethnic groups needed by the trial

The four recommendations are summarised in Fig. 1.

### Recommendation #1: Ensure your eligibility criteria and recruitment pathway do not limit participation in ways you do not intend

The trial eligibility criteria and recruitment pathway drive participant selection and trial teams need to consider how both might inadvertently affect the ability of different ethnic groups to take part in the trial. Most eligibility criteria target clinical characteristics but there are often non-clinical criteria too. These are usually linked to consent, language and ability or willingness to provide data, they are generally subjective and they are likely to disproportionally affect ethnic minorities. It is rare for trial teams to specify exactly *how* judgements are made around these criteria [13] (e.g. how language ability will be assessed), which leaves the decision down to recruiter discretion. Any eligibility criterion that relies on recruiter discretion is open to both conscious and unconscious bias against ethnic minority individuals (see *Recommendation #3*). The impact these non-clinical eligibility criteria might have on inclusion should be carefully considered and any potential negative effects (or the criterion itself) removed or mitigated. Finally, it is worth noting that clinical criteria may also exclude ethnic minority individuals because the disease presents differently or because diagnostic and other tests have not been designed with a range of ethnic groups in mind. For example, using pulse oximetry to warn of low blood oxygenation has for example been found to miss three times as many cases of occult hypoxaemia in Black patients as in White [14].

The recruitment pathway itself can introduce challenges to inclusion. Placing recruitment in, say, a hospital clinic makes an assumption that all potential beneficiaries of the trial intervention attend hospital clinics in the same way, which may be far from true. For example, some ethnic groups may be less likely to attend, or be referred to, those clinics, meaning individuals in these groups are, by design, less able to take part in the trial [15–17]. Using UK care homes to support recruitment to falls prevention trials is likely to disproportionately benefit White British people because a larger proportion of older adults from ethnic minorities are cared for in the family home [18]. Although it is beyond trial researchers to improve attendance in healthcare, steps can be taken to widen the recruitment settings. How people come into contact with the trial is an important consideration: trial teams need to consider the cultural and other preferences and beliefs that influence both health provision and care-seeking behaviour [17].

#### Examples

Some recruitment pathways may limit inclusion. For example, the ActWELL trial, which aimed to support reduction in breast cancer risk through weight loss and lifestyle changes for women aged 50 years or older in Scotland, identified participants using the Scottish Breast Screening Program [19], which is known to have differences in participation across ethnic groups (e.g. in 2002–2008 non-attendance for White Scottish women was 23% but 42% for Pakistani women) [20]. The trial then inherited these differences. The REFORM trial [21], which tested an intervention to reduce falls in older people, recruited through podiatry clinics and excluded patients who had attended high-risk clinics, (e.g. diabetes clinics). Participants who did not complete the baseline or run-in data collection instruments adequately or who were unable to read or speak English were also excluded. This resulted in a trial targeting older people that had a trial population where White British participants accounted for between 98.7 and 99.8% of all participants, close to 20% above 2011 UK census population level for White British people.

#### Public contributor comments

1. Consider placing recruitment in settings that people are familiar with or comfortable in: *‘Where you place your recruitment might be home turf.’*
2. It is not always clear why research is interesting or important to the communities: *‘..need to ensure that research is community targeted’.*
3. Depending on the trial, general practitioners (GPs) might be helpful for inclusive trials: ‘*If you engage with certain primary care groups who are dealing with specific conditions, you will bring in a diverse group. GPs know who to engage and can intercede on your behalf if you need further support. You’ve already got a trust relationship here so why not build on that?*’

#### Advice for good practice

1. Always ask the trial team whether an eligibility criterion may disproportionally affect the ability of one or more ethnic minority groups to take part in the trial [6].
2. Avoid subjective eligibility criteria particularly those based on language. Criteria can be made less subjective by specifying a concrete assessment or test to be used to support judgements.
3. Always check the anticipated match between the population with the condition of interest and the population of people the trial is likely to involve via the chosen recruitment pathway.

#### Practical resources

1. The NIHR INCLUDE Ethnicity Framework is a tool with a series of key questions to help trial teams consider the impact their trial eligibility criteria may have on the recruitment and retention of different ethnic groups. The tool is available at https://www.trialforge.org/trial-forge-centre/include/, along with a set of Frameworks that have been completed for real trials.
2. The Centre for Ethnic Health Research has developed resources to help researchers increase involvement of ethnic minority groups: https://ethnichealthresearch.org.uk/research/support-for-researchers/
  https://arc-em.nihr.ac.uk/clahrcs-store/equality-impact-assessment-eqia-toolkit
3. The United States Food and Drug Administration produced ‘Guidance for Industry’ in November 2020, which provides useful suggestions for how to ensure that eligibility criteria and trial design more generally do not restrict ethnic diversity: https://www.fda.gov/regulatory-information/search-fda-guidance-documents/enhancing-diversity-clinical-trial-populations-eligibility-criteria-enrollment-practices-and-trial
4. The Multi-Regional Clinical Trials Center of Brigham and Women’s Hospital and Harvard has guidance, tools and case studies that aim to improve diversity in clinical research: https://mrctcenter.org/diversity-in-clinical-research/

### Recommendation #2: Ensure your trial materials are developed with inclusion in mind

Trial materials include participant information leaflets, consent forms, materials linked to the trial intervention (e.g. information on how often to take a tablet) and outcome collection (e.g. keeping a sleep diary), as well as documents for safety reporting processes and results summaries given to participants. For this material to support inclusive recruitment and retention, trial teams need to consider language and whether that language is best written down, spoken, signed or expressed multimodally or in some other way. In instances where more than one language is used in the intervention materials, the trial team needs to consider whether the intervention is equivalent when offered in different languages.

The complexity of the above should not be under-estimated [22]. Early in trial planning, trial teams should carefully consider if translation into some participants’ dominant language(s) will be needed when it is not the majority or official language of society. In contexts where there is more than one official language (e.g. Wales), there may already be a requirement to offer materials in all official languages [23]. Effective translation means ensuring that language use is accessible, clear and appropriate, with cultural tailoring where needed. Materials should aim to be consistent and clear in all language versions and need to adhere to ethical requirements. Translation may also have implications for outcome assessment: measures may not be validated in more than a handful (or just one) language [24, 25]. Having made decisions at the trial planning stage around translation, whether this supports ethnic minority recruitment and retention as anticipated needs to be monitored.

Translation and interpretation can be complex and expensive. These costs are not only related to translation itself, but also to the increase in time and complexity of the work involved in developing and approving study documents. Robust back-translation (translating the translation back to the source language) will be required to validate the quality of translation [22]. Moreover, prior to translation, it is important to provide information regarding the context of the material so that the translation and back-translation process can take stock of this [24]. Consent processes that rely heavily on written material may present a substantial barrier to participation for some ethnic minority groups [26] and complementing written materials with multimedia, descriptive videos or illustrations can also help [27]. Other issues such as disability can also have an impact, so for example, individuals who are deaf may require sign language support. Video or audio information may be more appropriate if people would prefer to listen to information rather than read it. Some people will want to talk to someone who can answer questions and the main mode of information delivery becomes a conversation, with written and other information playing a much more supplementary role. It might also be worth considering ‘easy read’ versions of documents to cater to a wider range of lower literacy and English proficiency skills.

Where interpretation is needed, it should be provided by a professional interpreter or bilingual healthcare worker rather than a family member or friend because relatives or close ‘lay’ acquaintances may not be able to reliably interpret medical information or may withhold information that they share with the patient or offer an incomplete account or explanation [28]. In addition, the participant may not feel able to talk freely if confidentiality is not preserved [29]. However, if the participant would like a family member or friend present, including for additional language support, that wish should be respected. Approaches such as these ensure that individuals with low literacy, or who are unable to read/write in their own language, or who simply prefer to talk to someone about participation in a language they are more comfortable with can take part. Providing the option of verbal interpretation will sometimes be essential to ensure inclusion.

The importance of high-quality translation and interpretation is being recognised by approvals bodies. One of us (AMR) has observed in their research that all submissions to the UK’s Health Research Authority that explicitly state that there will be no translation of materials and that family members will be relied upon as interpreters will be asked to justify these decisions. Approval will be denied if this justification is found wanting.

#### Examples

In a qualitative study targeting South Asians, the participant information sheet and consent forms were translated into Urdu and Gujarati using professional translation services [30]. The materials were then back-translated by a bilingual researcher and a member of a local community group to ensure that the information was culturally appropriate with conceptual equivalents and that the translation was easy to read and understand. This helped recruit nine of the 27 participants, including six Asian/Asian British Pakistani women of varying age groups and three older Asian/Asian British Indian participants. Of the six Asian/Asian British Pakistani women, four had no previous experience of taking part in research. All four said they participated because the researcher had approached the women through their community group organiser (see *Recommendation #4*).

Community engagement work done in the Kilifi District, just outside Mombasa, Kenya [31] found that in Kigiriama and Kiswahili—the local languages—there are no equivalent, widely understood, terms for Western concepts of ‘research’. Terms such as ‘*utafiti*’ and ‘*uchunguzi*’ were synonymous with ‘investigation’ or ‘test’ for clinical treatment. Although done in Kenya, translation work in the UK that used these languages would have to tackle conceptual differences between English and Kigiriama and Kiswahili with regards to ‘research’ before getting to the trial itself.

##### Public contributor comments

1. ‘*Involve people from minority ethnic groups in a patient and public involvement capacity*.’
2. Translation is not just about the world language: *‘Language used is simple so that it is more inclusive- health literacy’; ‘Avoid jargon’; ‘Empower everyone to participate’.*
3. It is important to consider the language ability of the clinical team: *‘Not just multilingual researchers but also clinical team to be multilingual.’*
4. The people doing translation need to be fluent: *‘Person should be from related backgrounds and should be fluent in specific language that is to be translated.’*
5. Consider ways to communicate: *‘Verbal face-to-face communication important. Think about how best to actively communicate the information verbally. People don’t [always] read in their own language.*’
6. There is a need for openness and transparency in the information provided: ‘How are they going to use the information, privacy, confidentiality etc. Harms and benefits from research. Useful to help decide whether to take part or not.

##### Advice for good practice

1. Consider language proficiency (in English and other languages) of the target ethnic groups very early in trial conceptualisation [22].
2. Translation should use robust methods (e.g. forward and back translation with input from multiple translators and clinical reviewers) with the aim of achieving conceptual equivalence [22].
3. Inclusion needs to be considered at all stages of the trial process [22].
4. When planning budgets in funding applications for future trials, ensure sufficient resources are requested for translation.

##### Practical resources

1. Write documents in clear, simple language to support participation of people from all ethnic groups. The US Centers for Disease Control and Prevention gives useful advice at: https://stacks.cdc.gov/view/cdc/11938.
2. Examples of translation and interpretation budgets, plus additional guidance for when working with interpreter are given in Supplementary File 1.
3. The American Medical Association has a guide to communicating with patients with limited proficiency in English, including advising on using different types of interpreters and which has relevance to trials. Table 1 is especially useful: http://onlineresources.wnylc.net/pb/orcdocs/LARC_Resources/LEPTopics/HC/2008_AMA_OfficeGuidetoLEPPAtientCare.pdf.
4. The European Centre for Disease Prevention and Control has a useful guide on cultural adaptation of health communication materials, including examples: https://www.ecdc.europa.eu/sites/default/files/media/en/publications/Publications/translation-is-not-enough.pdf.
5. The Centre for Ethnic Health Research provides professional translation and cultural adaptation services: https://ethnichealthresearch.org.uk/services/translation-cultural-adaptation/.
6. Community organisations (e.g. Grampian Regional Equality Council; https://grec.co.uk) might be able to assist with translations or back-translations. Keep in mind Table 1 in the American Medical Association guide (see above) if the organisation does not use professional translators and interpreters.
7. NIHR suggests checking with your organisation for approved vendors for translation service and if none are available exploring providers like thebigword (https://en-gb.thebigword.com) or OSW (http://www.subtitling-uk.com/translation/).

### Recommendation #3: Ensure trial staff are culturally competent

All trial staff should have cultural competency training that raises awareness of the need to involve participants from ethnic minority groups, dispels incorrect cultural stereotypes and increases staff confidence in talking to people with different ethnicities to their own [10, 32].

A common recruitment approach is for a clinical or research staff member to directly approach potential participants in a clinic or waiting room to ask if they would be interested in taking part in the trial. The respective cultural perspectives of the member of staff and the potential participant can lead to uncertainty and misunderstandings [17]. Those tasked with recruitment may be hesitant, unwilling or not feel confidently trained to raise trial participation with people from different ethnic groups. The reasons behind this may include fear of inadvertently causing offence, assuming that a person will not be interested, a belief that the person will be more difficult to recruit and retain, assuming that the person will not have ‘good’ English, and conscious or subconscious bias, including assuming that the trial will neither benefit or be acceptable to someone from a particular ethnic group. There may also be fundamental differences in the concept of consent. For example, individual consent can be at odds with Chinese ethical traditions of wider family involvement in decisions about healthcare [33].

Each of us has our own attitudes and beliefs and these may affect our willingness to participate in a trial. Overall, ethnic minorities are as likely as majority populations to consent to take part in a trial if they are offered the opportunity to participate [34, 35]. Trial staff involved with recruitment, retention and other trial procedures therefore need cultural competency training to ensure that this offer is appropriately made. Training will highlight the need to involve people from different ethnic groups in the trial, the potential barriers to achieving this and give staff the confidence and skills to discuss the trial with people of all ethnicities [17]. Such training will also be useful for staff not directly involved with participants (e.g. methodologists) because it will raise awareness of issues that need to be accounted for in design (see *Recommendation #1*).

#### Examples

A recent systematic review of strategies to recruit ethnic minority adults to UK clinical trials found that 11 of the 21 trials specifically reported having recruitment staff from the same cultural and ethnic background as potential participants and four trials where female staff approached female participants [17]. Such staff may also be multi-lingual, which could help minimise language barriers (see *Recommendation #2*). Care is needed though: the effectiveness of this strategy was not evaluated [17] and being from a particular ethnic group does not mean staff are guaranteed to have cultural competence [36].

Patient and public involvement and working with community organisations during trial design can help to make trial procedures more inclusive. For example, one of the authors (SD) working with three South Asian public contributors as part of recruitment planning for an in-vitro fertilisation trial found that these women (all with lived experience of in-vitro fertilisation) wanted to discuss the details of any future trial with a woman but one who was *not* of South Asian ethnicity (unpublished work). Infertility carries a stigma in South Asian culture. These women were worried that discussing the trial with a South Asian staff member risked details of their infertility ‘leaking’ into the local community.

##### Public contributor comments

1. ‘*Researchers’ lack confidence to approach people*’. This might be down to language, or difficulty relating to other cultures. Think about your team: ‘*Research team made up of ethnic minority researchers or patient reps that people can identify themselves with*’; ‘*Seeing professionals who are the same as us, trying to create solutions and cures, will build more trust*’.
2. ‘People make a lot of assumptions based on ability to communicate or cognition etc. Educate researchers on how to approach people. How you engage with them is really important and something to consider ‘.
3. Language is important: ‘*Not all terms are relevant. Need to discuss what terminologies are acceptable*’.
4. Including ethnic minority community members in staff training may be helpful: ‘*How about training recruiters and community groups together in approaching people for clinical trials where quick consent is required?*’

##### Advice for good practice

1. All trial staff should have cultural competency training [10].
2. Depending on the trial and the ethnic groups that must be involved, training could focus, or be more detailed, for some ethnic groups than others.

##### Practical resources

1. The Centre for Ethnic Health Research in the UK is currently running courses on cultural competence: https://centreforbmehealth.org.uk/training-courses/cultural-competence/.
2. Vocal https://www.wearevocal.org/) and the Black, Asian and Minority Ethnic Research Advisory Group (BRAG; https://www.wearevocal.org/opportunities/black-asian-and-minority-ethnic-research-advisory-group-brag/) have co-created a free short self-taught course on inclusive research https://catalogue.manchester.ac.uk/browse/i3hs/open-courses/courses/inclusive-research.
3. The NIHR INCLUDE online training provides information and support on how to improve the inclusion of under-served groups in health research: https://newcastlejro.com/2021/04/nihr-include-online-course/?utm_source=rss&utm_medium=rss&utm_campaign=nihr-include-online-course.
4. There is a video from NIHR about cultural competence in research: https://www.youtube.com/watch?v=_VEibZOP01c&t=48s. This is part of a series of videos from the NIHR about ethnic diversity in research: https://www.youtube.com/playlist?list=PLIa1oelW_zJ_3wXmB9nVAReTFQSR5GTiY.

### Recommendation #4: Build trusting partnerships with community organisations that work with ethnic minority groups

Taking part in a trial needs trust and generally research institutions and researchers have previously not engaged adequately to build trust with ethnic minority groups [37]. Lack of trust has for example been highlighted as an important factor driving recruitment of ethnic minority participants to COVID-19 vaccine trials [38] and to vaccine uptake itself [39].

Trust is not built quickly. A good place for trial teams to start is by building relationships with local community organisations (including faith groups) working with ethnic minority groups in their area [10, 40]. These organisations are more likely to have the trust, skills and cultural awareness to engage with members of the communities they serve and can facilitate discussion between those individuals and researchers. Community organisations should be involved from the beginning of trial design so that members’ views can have a meaningful impact on design decisions and once involved, trial teams need their continued advice throughout the trial. However, rather than a focus on building trust *in* ethnic minority groups, the responsibility lies with individuals and research institutions to enhance their trustworthiness and so create the conditions for trust to flourish [41].

#### Examples

The Culturally Adapted Family Intervention (CaFI) study [42] aims to improve the treatment of schizophrenia and psychosis for people from African and Caribbean backgrounds. Members of the African and Caribbean community have been actively involved in the study from the beginning using an approach that emphasises equality between community and academic partners in identifying problems, devising, implementing and evaluating solutions and disseminating findings. Community members who expressed interest received honorary university contracts enabling access to academic resources including research methods training to facilitate capacity building and development of future research to benefit the community.

Work with community groups is a form of patient and public involvement (PPI) and as with PPI elsewhere in healthcare, it is important that community group members be reimbursed for their contributions. For example, Tierney and colleagues [43] worked with two community group organisers who acted as interpreters during a workshop to help identify research priorities for South Asian women. The two group organisers were paid for their time at a rate similar to the 2021 UK NIHR rate for PPI contributors of £25 per hour [44]. Budgeting to pay for room hire in community spaces and catering for meetings are other ways to invest back in community groups and illustrate that you value their input.

##### Public contributor comments

1. There is a lack of trust in the research process and researchers: ‘*Researcher are not known to members so on the first meeting it is less likely to build the trust in a first instant‘; ‘Need to come to the community and raise awareness about the trial face-to-face’; ‘What are the key components that people in each community find difficult with the health sector? e.g.: historical ‘trials*’ which certain communities don't forget. Mistrust can be passed down through generations.
2. People never hear what happened in the research: ‘*Results of issue never communicated. Lack of feedback*’.
3. Outreach work with communities is likely to be beneficial: ‘*Specified work - e.g.: outreach in schools and colleges educating younger people about the whole process of trials through to end stage will help people understand how it actually works. e.g.: community work in older communities who aren't familiar with jargon, tech and innovations in medical and associated fields. It isn't education as such as engagement, involvement and participation*’.

##### Advice for good practice

1. Start building relationships with local community organisations that work with ethnic minority groups. Don’t think of this as a transactional, trial-specific activity but as an ongoing long-term relationship.
2. Your collaboration with community organisations and ethnic minority public contributors should start early in the life of the trial.
3. Community organisations cannot work for free. Ensure your grant has a realistic budget to support collaboration.
4. Organisations involved in research (e.g. universities) should promote their trustworthiness by ensuring ethical research conduct and engaging with stakeholders to help promote the social value of research [37]. This can help to overcome the stigma and mistrust associated with the research process [17].

##### Practical resources

1. Organisations that can provide links to ethnic minority public contributors and community organisations that work with ethnic minorities include:

- The Manchester Black, Asian and Minority Ethnic Research Advisory Group: https://www.wearevocal.org/opportunities/black-asian-and-minority-ethnic-research-advisory-group-brag/.
- Egality Health: https://www.egality.health.
- The Centre for Ethnic Health Research: https://ethnichealthresearch.org.uk.
- Faith Action: https://www.faithaction.net.
- Advertise your study for PPI contributors via NIHR People in Research: https://www.peopleinresearch.org. You can access local community groups via Voluntary, Community and Social Enterprise (e.g. Community Healthcare Organisations, Health Service Executive, Ireland https://www.theatnetwork.com/community-healthcare-organisations-in-ireland/)

2. NIHR has produced comprehensive guidance for how to budget for patient and public involvement in research: https://www.nihr.ac.uk/documents/payment-guidance-for-researchers-and-professionals/27392. A guidance to consider when paying people who are on benefits: https://www.scie.org.uk/co-production/supporting/paying-people-who-receive-benefits.
3. In England and Wales, the local Research Design Service should have links to community groups and may also offer funding for pre-grant public involvement. An example is Yorkshire and Humber: https://www.rds-yh.nihr.ac.uk/public-involvement/database/.

## Discussion

Ethnic minorities in the UK and elsewhere are under-represented in randomised trials. Trials that ignore or forget groups of people who could gain benefit from the intervention under test not only contribute to inequality, they are bad science [45–47]. This is especially true for pragmatic, practice-focused trials intended to inform clinical policy. Creating policy around trials that are not inclusive runs the risk of believing we have solved a healthcare problem for all when in fact things have only got better for some. In short, trials that fail to account for the needs and perspectives of ethnic minority individuals in their design, conduct and reporting are likely to widen, not narrow, health inequalities.

We have presented four recommendations that we think trial teams need to follow if they are to successfully involve ethnic minority individuals in their trials. Different ethnic minority groups have different needs and strategies, which means implementation of the recommendations will vary depending on the needs of the particular target population. While there may be similarities between the strategies used to support inclusion of different ethnic minority groups, it is unlikely that there is any strategy that will be equally effective for all ethnic groups. Some strategies may work well for one group but be completely ineffective for others. Ethnic minority groups are not homogeneous [6]. There are other things trial teams can consider including accounting for multiple and complementary strategies to improve inclusion (see for example Bodicoat et al. [10]) but the four recommendations we propose are an important and realistic place to begin.

Moreover, we think that acting on these recommendations will influence recruitment *and* retention. For example, community-based recruitment approaches have been found to also improve retention [48–51]. PPI contributors from relevant communities are central to all our recommendations and building and sustaining trust and relationships with them and the communities they represent are key for long-term impact on trial participation. Tailored approaches that are designed to be inclusive (e.g. collecting data through multiple means including face-to-face, emails, text messages, online, or using a range of methods to tell people about trials) are likely to help people feel more able to take part in a trial, and to then stay involved.

Finally, by providing public contributor comments with our recommendations, we hope that readers will understand why these recommendations are important and we hope the examples, advice for good practice and practical resources will make it easier for the recommendations to be implemented. Implementation of the recommendations will, however, need money and time, which funders, grant reviewers, approvals bodies and trial teams themselves need to recognise.

This guidance is intended to give practical support to trial teams to help them to recruit and retain trial populations that more closely resemble the population that would gain benefit from an effective treatment or initiative. There is some research on the factors that influence trial recruitment and retention of ethnic minority individuals [10, 24–37, 40], but those working in trials are greatly limited by the lack of empirical evidence for effective strategies to mitigate these factors. It is a sad fact that at the time of writing there is no strategy in the Cochrane recruitment review [7] or the Cochrane retention review [8] that would definitely help a trial team more effectively recruit and retain ethnic minority participants. There are in fact few evidence-based recruitment and retention strategies to turn to full-stop, let alone ones aimed at recruiting and retaining ethnic minority participants.

We encourage all those working in trials, ourselves included, to do more evaluation of the strategies and approaches we use to improve trial inclusion. Studies Within A Trial (SWATs) [52] are one way to do this but evaluation of all stripes, including qualitative work (which itself could be a SWAT), would be most welcome. The Trial Forge team would be happy to help where we can: get in touch at info@trialforge.org.

In a project looking at factors affecting COVID-19 vaccine uptake by ethnic minority groups [39], one community organisation contributor made the point that “change was not about reprogramming ethnic minorities but about reprogramming organisations”. The organisations alluded to were the UK Government and the NHS but the point applies equally well to those of us who design, run and report randomised trials. It is we who need to change; it is our behaviour that needs to be different. If recruitment and retention of ethnic minority individuals in randomised trials does not improve it will be our fault, no-one else’s.

## Supplementary Information

**Additional file 1.**

## Data Availability

Not applicable for this article.

## Abbreviations

COVID: Coronavirus
GP: General practitioner
INCLUDE: Innovations in Clinical Trial Design and Delivery for underserved groups
NHS: National Health Service
NIHR: National Institute for Health and Care Research
PPI: Patient and public involvement
SWAT: Study Within A Trial
UK: United Kingdom
