# Correction: Trial Forge Guidance 3: randomised trials and how to recruit and retain individuals from ethnic minority groups—practical guidance to support better practice

**DOI:** 10.1186/s13063-022-06669-z

**Published:** 2022-09-07

**Authors:** Shoba Dawson, Katie Banister, Katie Biggs, Seonaidh Cotton, Declan Devane, Heidi Gardner, Katie Gillies, Gosala Gopalakrishnan, Talia Isaacs, Kamlesh Khunti, Alistair Nichol, Adwoa Parker, Amy M. Russell, Victoria Shepherd, Frances Shiely, Gillian Shorter, Bella Starling, Hywel Williams, Andrew Willis, Miles D. Witham, Shaun Treweek

**Affiliations:** 1 Centre for Academic Primary Care, Bristol Medical School, University of Bristol, Bristol, BS8 2PS UK; 2 Health Services Research Unit, University of Aberdeen, Aberdeen, AB25 2ZD UK; 3 School of Health and Related Research, University of Shefeld, Shefeld, S1 4DA UK; 4 Health Research Board-Trials Methodology Research Network (HRB-TMRN), School of Nursing and Midwifery, National University of Ireland Galway, University Road, Galway, Ireland; 5 Department of Surgery and Cancer, Imperial College London, London, W12 0NN UK; 6 UCL Centre for Applied Linguistics, IOE, UCL’s Faculty of Education and Society, University College London, London, WC1H 0AL UK; 7 Diabetes Research Centre, College of Life Sciences, University of Leicester, Leicester General Hospital, Leicester, LE5 4PW UK; 8 National Institute for Health Research (NIHR), Applied Research Collaboration (ARC) East Midlands, University of Leicester, Leicester, UK; 9 School of Medicine, University College Dublin, Dublin, Ireland; 10 York Clinical Trials Unit, University of York, York, UK; 11 WHO Disability Team, Geneva/ Leeds Institute of Health Sciences, University of Leeds, Leeds, UK; 12 Centre for Trials Research, Cardif University, Neuadd Meirionnydd, Heath Park, Cardif, CF14 4YS UK; 13 Health Research Board Clinical Research Facility and School of Public Health, University College Cork, Cork, Ireland; 14 Drug and Alcohol Research Network, Queen’s University Belfast, Belfast, UK; 15 Centre for Improving Health Related Quality of Life, School of Psychology, Queen’s University Belfast, Belfast, UK; 16 Public Programmes Team (now Vocal), Manchester University NHS Foundation Trust, Research & Innovation Division, The Nowgen Centre, 29 Grafton Street, Manchester, M13 9WU UK; 17 NIHR Manchester Biomedical Research Centre, NIHR Manchester Clinical Research Facility, Manchester, UK; 18 Centre of Evidence-Based Dermatology, Queen’s Medical Centre, Nottingham University Hospitals NHS Trust, Nottingham, NG7 2UH UK; 19 NIHR ARC East Midlands, University of Leicester, Leicester, UK; 20 NIHR Newcastle Biomedical Research Centre, Campus for Ageing and Vitality, Newcastle University and Newcastle upon Tyne NHS Trust, Newcastle, NE4 5PL UK

**Correction: Trials 23, 672 (2022)**

**https://doi.org/10.1186/s13063-022-06553-w**

Following the publication of the original article [1], we were notified of a spelling mistake in the 5th author’s name. “Declane Devane” should be “Declan Devane”.

The original article has been corrected.
