# Supplementary material for: Trial Forge Guidance 3: randomised trials and how to recruit and retain individuals from ethnic minority groups—practical guidance to support better practice
Source: Trials. 2022 Aug 17;23:672. doi: 10.1186/s13063-022-06553-w (PMC9383663; doi:10.1186/s13063-022-06553-w)
Supplement: Supplementary file 1 — Additional file 1. [file 13063_2022_6553_MOESM1_ESM.pdf]

Questions to think about when planning translation

(This text and examples were provided in January 2022 by Rosilda Panoni, In-Trans Coordinator at Grampian Regional Equality Council, UK (http://in-trans.co.uk)).

Here are some questions that may help a trial team think through their translation needs before speaking to a translation organisation:

Identify your needs

- How important is multilingual and communication for your project or organisation?
- Do you require face to face interpretation, translation of written text, or both?
- How many languages do I need to translate into?
- Try to get information about the language's distribution in your target area and consider that alongside the focus of your project.

Translation project:

- Which material should be translated first?
- Which target languages do you want to prioritise? Is it critical to translate all content into all relevant languages at the beginning?
- For example: You want to translate a questionnaire into Latvian, Lithuanian, Russian and Polish. Since Latvians and Lithuanians very often speak Russian, you might consider first translating the questionnaire into Russian and monitoring the requests for Latvian and Lithuanian, perhaps adding a line on the Russian translation about the possibility of translating the material into Latvian and Lithuanian. A good quality translation service will provide you this advice if you arrange a discussion in the planning stages of your project.
- Another example will be, translate questionnaires first and check the responses and interaction of your audience before starting to translate reports or other support materials.
- It may be helpful to get a sense of the language demand before going ahead with translation, e.g., by speaking to community contacts about the likelihood of community take up and whether the English version would suffice. If the demand is low, maybe the

option to get an interpreter to attend a focus group session to discuss the content of the report would be another option.

- In some communities, members will only *speak* the language and will be not able to read. In situations like this getting an interpreter to read the report will also be a solution.

Optimise your files

- Send the original editable source files, in this way you will avoid cost of formatting that could increase the cost of your translation.
- Some languages (e.g., Arabic, Mandarin, Cantonese, Hindi) will require different setting or formatting

Interpreting project:

- Which target languages do you want to prioritise?
- When will the sessions take place?
- How many participants do you intend to have?
- Do you want to have one-to-one interviews or a group discussion?
- When you are using interpreters, you need to think that you will need at least double the time of an interview without an interpreter. You will need to allow time for the interpreter to do his/he job.
- It will be a good practice to create a poll to establish the number of participants which will attend the event and the languages required
- The location also could have an influence on the budget. If the session will be delivered out to the city, travel costs may occur. The agency will try to get the closest interpreters to attend the session. So, maybe it will be better to ask for a quote based on the location of the appointment. The agency will advise of the options available.

Budgeting

- Identify the financial resources available for your project.
- Get a quote or some idea about the cost involved and make sure that you included the money for translation and interpreting support in your funding requests.
- Establish a timeline, never leave translation to the last minute. This could increase the cost of the translation.

- Think about the location for the group discussion or interviews. If remote meetings using video are appropriate, this will cut the costs.

Example costs for using In-trans at Grampian Regional Equality Council

Follow below our rates for interpreting and translations jobs.

Translation:

Cost of translation is based in the number of English words. Translation above 200 words:
For languages Group 1 (Polish, Russian, Portuguese, Lithuania, etc) will be £0.10p per word plus VAT
For languages Group 2 (Arabic, Hindi, Mandarin, Cantonese, etc) will be £0.12p per word plus VAT

Translation under 200 words will be charged £30.00

Formatting will be charged £30.00 per hour (if necessary)
Proof of reading will be charged £0.08 per word (if necessary).

Interpreting:

The rate of interpreting is £30.00+ VAT for up to one hour during office hours (8:00-17:00) and after the first hour by the minute.

There is no travel fee in the cases below:

- in case we are able to provide an interpreter locally
- travel need is less than 10 miles one way (20 miles in return)

In other cases, travel expenses occur. We always inform you about it in advance, so that you can decide if you pay for the travel or prefer to change the date/ time of the appointment to have an interpreter locally.

The charge of travelling time is £18.00 up to one hour and any time over one hour will be charged on a minute basis. The travel rate per mile is £0.48p

Cancellation will be accepted at any time. However, by our policies, cancellations occurring 24 hours before the appointment will

be charged for the first hour. An appointment cancelled over the 24 hours before the appointment will not be charged.

Example costings

These examples were provided in January 2022 by Dr Andrew Willis, Centre for Ethnic Health Research, University of Leicester, UK.

Example costing 1. Animated recruitment video

Background

Production of a 2-3 minute recruitment animated video to support recruitment of participants with lower levels of English literacy to a large national trial taking place in an acute hospital setting. The animation and voiceover were co-designed and produced by patients and carers from ethnic minority communities during 3 workshops facilitated by designers and trial researchers.

The team followed an established methodology for translation to maintain content validity, including clinician review to check clinical accuracy(1). The video was developed by an in-house design team using Vyond software (https://www.vyond.com). Translation of the script was done in-house, with two translators for each language, one of whom recorded the voiceover.

Item	Cost
Co design workshops x 3, Video storyboarding, production and refinement, review of final version.	£2500
Production of 2-3 min animated video in English, 2 stage translation dubbed into 4 languages (Polish, Urdu, Bengali and Punjabi) with pilot testing.	£3000
Training sessions to cover video dissemination within the main trial	£750
**Total cost**	**£6250**

Example costing 2. Questionnaire translation

Background

Translation of a (Approximately 2000 words) paper based self-assessment risk score for type 2 diabetes from English into Urdu. The research team followed an established translational model of firstly simplifying the English version and development of conceptual guidance for translators to follow.

This was followed by forwards translation from English to Urdu using two qualified translators and then back translation from English to Urdu. Translators had to hold a diploma in public service interpreting and they were recruited from different translation companies locally.

Once all translators were happy with the final version, it was reviewed by two Urdu literate clinicians and piloted with a group of 10-15 community members to check reading age, level of understanding and ease of use.

Item	Cost

Researcher salary costs (recruitment, project management.	£1750
Payments for translators.	£2250
Community member/clinician vouchers	£250
Design costs	£750
**Total cost**	**£5000**

1. Patel N, Willis A, Stone M, Barber S, Gray L, Davies M, et al. Developing a conceptually equivalent type 2 diabetes risk score for Indian Gujaratis in the UK. Journal of diabetes research. 2016;2016.

A GUIDELINE TO WORK WITH INTERPRETERS

Interpreting is a two way process. Our aim is to ensure professionals can adopt good practice and have the skills required to use interpreting and translation resources appropriately.

Why you should use interpreters

Using a professional interpreter is particularly important when communicating with people with little or no English language to ensure that you can offer:

- **Neutrality**: although relatives, friends and neighbours can be capable interpreters, they may take on an advocacy role rather than being objective and neutral when communicating your client's ideas or information.
- **Confidentiality**: a client may not be happy to disclose accurate, detailed or relevant information in front of a person doing the role of interpreter if they know the person outside the interpreting setting. Clients bringing their children to interpret offer an additional risk; in addition to more chances of getting incorrect information, this could be inappropriate use of a minor.
- **Compliance**: professional interpreters' skills and language competence are assessed and standardised. Their capability to interpret fluently and accurately can save you time and ensure that your client understands what you are communicating. Interpreters respect the clients' rights and uphold their obligation to ensure that service users and their clients can follow standard procedures in spite of the language barrier.

What is the role of the interpreter

The interpreter's aim is to facilitate successful communication over linguistic and cultural boundaries. The interpreter is an essential link between the communicating parties helping them to express to the full extent of their mother tongue.

Interpreters may belong to one of these two categories:

- **Qualified interpreters**: They may hold a DPSI qualification (Diploma in Public Service Interpreting) with a particular emphasis on areas related with other experience such as health, law, local government, etc which reinforces their knowledge of specific vocabulary in their area of expertise.
- **Community or Liaison Interpreters**: they are usually bilinguals with high command of both languages and cultures and have passed the screening criteria of a particular organization and have been awarded a certain recognition. They facilitate communication in public services such as healthcare or community services and in settings such as government agencies, community centres, legal settings, educational institutions, and social services.

What you can expect from an interpreter

Interpreters are bound by their code of practice and confidentiality, therefore they remain impartial and unbiased towards the information communicated by all parties involved. This ensures that your message is conveyed accurately and that your client's information is protected and translated to the best of the interpreter's ability.

How to work effectively with interpreters

The following points will ensure good practice when using a professional interpreter:

- Ask the interpreter to arrive 5 minutes before your client. Provide a brief about the case and choose the kind of interpreting style you want to use (consecutive, simultaneous or whispering)
- Allow enough time for the interview, keeping in mind that when using an interpreter you will need more time with your client.
- Never leave the interpreter alone with your client. Your client may establish a relationship with the interpreter rather than with you or could disclose information the interpreter may fail to disclose later.
- Use the triangle position as a sit arrangement. Face your client during conversation to strengthen the relationship you are establishing with the client. Wherever possible, the interpreter should sit at an even distance between you and your client.
- Don't use jargon and clarify in advance the meaning of any acronym you need the interpreter to use or explain to your client.
- Make sure that you allow enough time for the interpreter to convey your message to the client in their mother tongue.
- Maintain control of the appointment at all times. Don't assume that someone understood what you said because they nodded their head or said "yes". If you become aware of any problem with communication, your client appears uncomfortable or if the interpreter is struggling, stop the meeting and have a break. Assess the situation and continue if appropriate.
- Be aware that there may be cultural differences in non-verbal communication. E.g. Gestures that indicate politeness, eye contact etc.
- If for any reason you are called away from the appointment invite the interpreter to come with you.
- Take time to debrief the interpreter at the end of the appointment. Five minutes debrief once the client has left will give you the opportunity to clarify any doubt you may have about the discussion.
- Complete and sign the interpreter's form
- If a follow up meeting is required contact your service provider and request the same interpreter if relevant

Interpretation types

1. **Simultaneous interpreting:** most commonly used in international level meetings and court settings, it is real-time interpreting which takes place while the original speaker communicates. It typically involves the interpreter sitting in a sound proof booth with a microphone. Listeners hear the simultaneous translation via headphones. A variation of this setting is called **whispering** and it involves the interpreter sitting next to the person requiring translation so that it also takes place in real time while the interpreter whispers to the client while the message is delivered.

2. **Consecutive interpreting:** the speaker pauses to allow the interpreter to repeat what has been said in the target language before continuing. Consecutive interpreting is very popular because it does not require any equipment or complex planning. It does not happen in true real-time as the speaker must pause to give the interpreter time to speak.

How to book an interpreter

- Complete the form with all relevant information about the appointment :
  - Date
  - Time –*make provision for additional time required to translate your message and that of your client*.
  - Location
    - ***Home visits*** *– Interpreters are instructed to wait outside of the property where the appointment will take place. You must ensure that you provide a mobile phone to the service provider in case there is any need to contact you due to unforeseen circumstances.*
  - Check that the language required is correct
  - Check for any specific requirements. E.g: Female interpreter
- Send your form to your service provider in advance to ensure that there is enough time to arrange an interpreter to attend.
- When you receive the booking confirmation, keep the name of the interpreter that will attend the appointment and inform reception or any other relevant party involved on the date of the appointment.

Example interpretation costings

These examples were provided in January 2022 by Dr Talia Isaacs, UCL Centre for Applied Linguistics, IOE, UCL's Faculty of Education and Society, University College London, UK.

The costs below are based on the following request from Talia to several companies:

'Our 1-hour data collection session will consist of going over translated informed consent materials (consent form & information sheet) and then conducting semi-structured one-on-one online interviews with South Asian patients who have been invited to participate in either diabetes or depression trials.

Do you have professional interpreters for East Asian languages? If so, could you send me a quote for one hour of the interpreters to me?'

Response from two companies

Company 1

£40-£55 for interpretation for a 1-hour remote data collection session, consisting of informed consent + semi-structured interview.

In-person requires 2-3 hours of work + travel costs

Translation is £125-£145 per 1000 words. Transcription is 75 p per minute = £40 – This quote excludes timecode and strict verbatim (e.g., filled pauses). Including those for better transcription fidelity would be more expensive.

Rough total for 50-minute interview (excluding transcription of informed consent process): Presuming 5000-8000 word responses that need to be translated for interview

Range of quote (presuming lowest and highest rates with the non-verbatim transcription cost)**:** £703 - £1,253

Company 2

£120-£160 for interpreta6on for a 1-hour remote data collection session.
